# Supplementary material for: Molecular Mechanisms of Gain-of-Function Mutations in λ Cro Revealed by Molecular Dynamics Simulations
Source: ACS Phys Chem Au. 2025 Oct 21;5(6):716–28. doi: 10.1021/acsphyschemau.5c00082 (PMC12670291; doi:10.1021/acsphyschemau.5c00082)
Supplement: Supplementary file 1 [file pg5c00082_si_001.pdf]

Supporting Information:

Molecular Mechanisms of Gain-of-Function Mutations in λ Cro Revealed by Molecular Dynamics Simulations

Ryan Hebert,[†,§] Alexander Perez[†,§], and Jeff Wereszczynski[*,¶,§]

[†]Department of Physics, Illinois Institute of Technology, Chicago, IL 60625, USA

[¶]Departments of Physics and Biology, Illinois Institute of Technology, Chicago, IL 60625, USA

[§]Center for Molecular Study of Condensed Soft Matter, Illinois Institute of Technology, Chicago, IL 60625, USA

email: jwereszc@illinoistech.edu

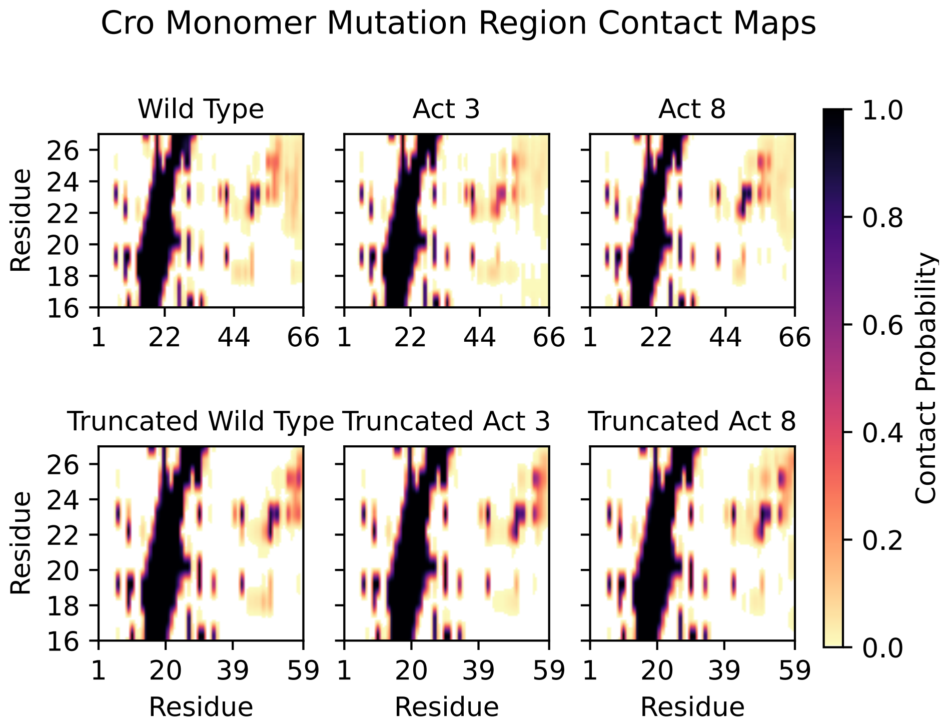

Figure S1: Contact maps of the mutation region of Cro monomers against the entire monomer.

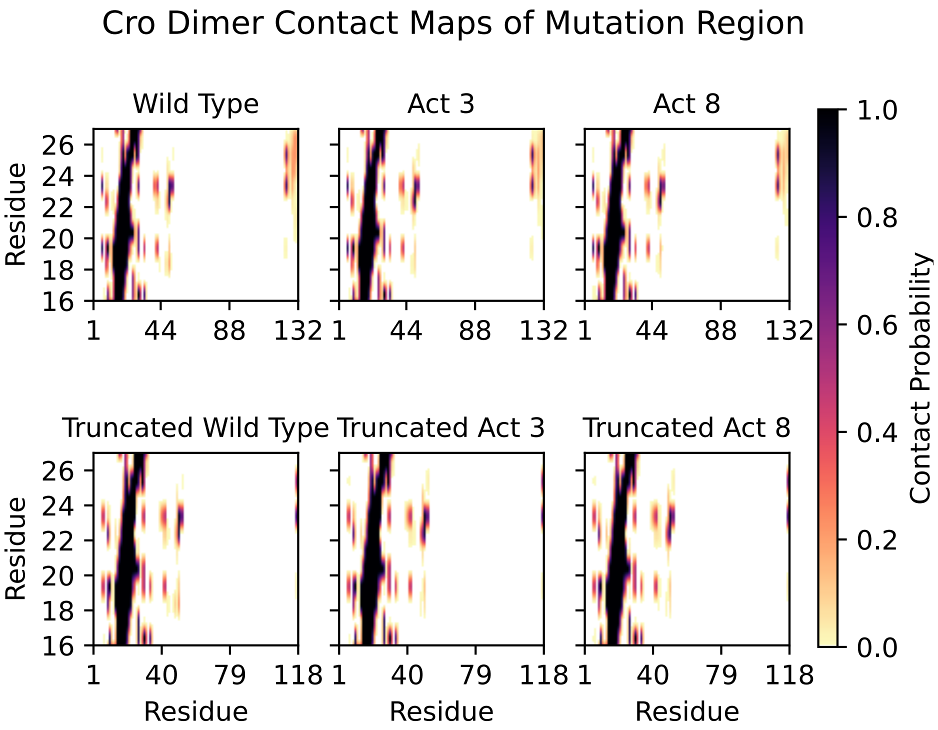

Figure S2: Contact maps of the mutation region of the first subunit of the Cro dimer against the dimer complex. Full contact maps of the dimer complex are given in S5 and demonstrate a similar contact pattern across subunits, differing largely by the distribution of C-terminal contacts.

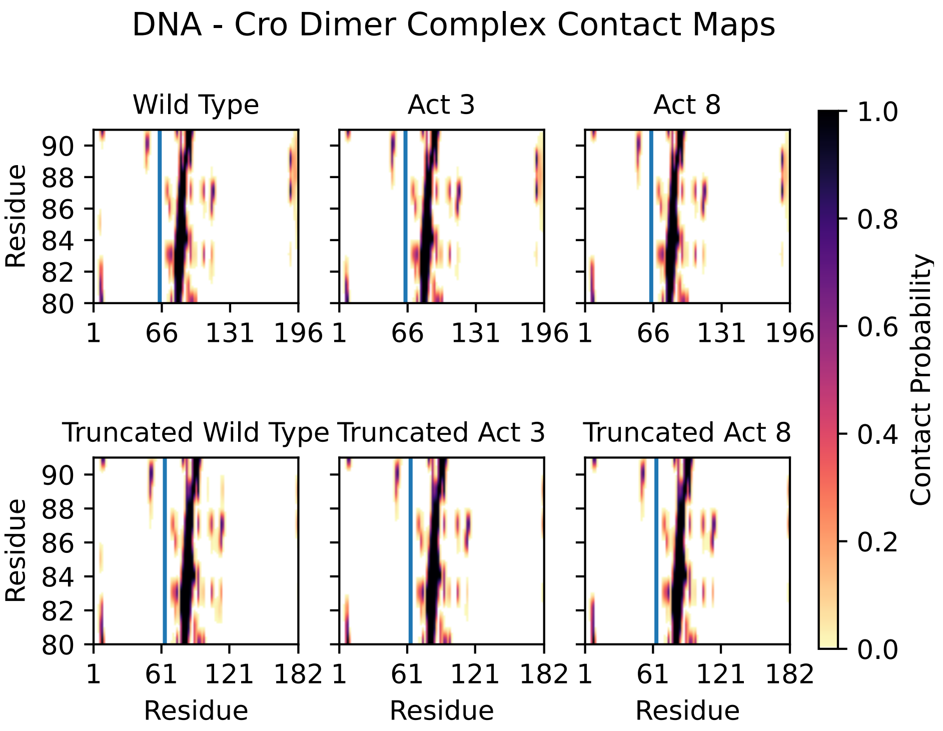

Figure S3: Contact maps of the mutation region of the first subunit of the Cro dimer against the DNA-dimer complex. Residues 1-64 represent DNA. Full contact maps of the DNA-dimer complex are given in S6 and demonstrate a similar contact pattern across subunits, differing largely by the distribution of C-terminal contacts.

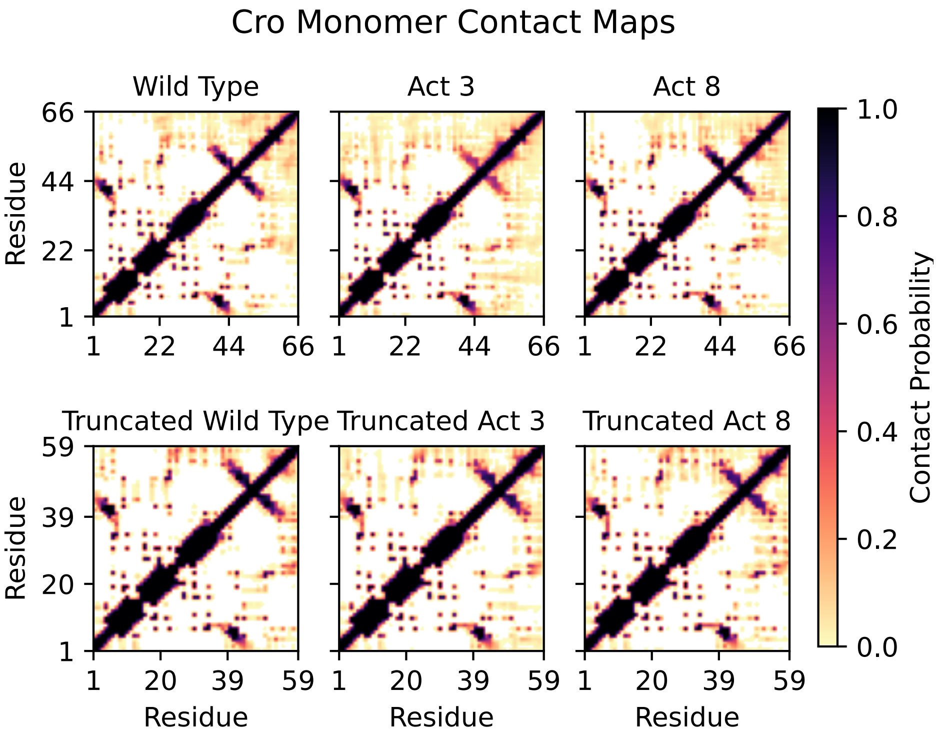

Figure S4: Contact maps of Cro monomer structures. The final βstrand is prone to unfolding, smearing out contacts from the C-terminus.

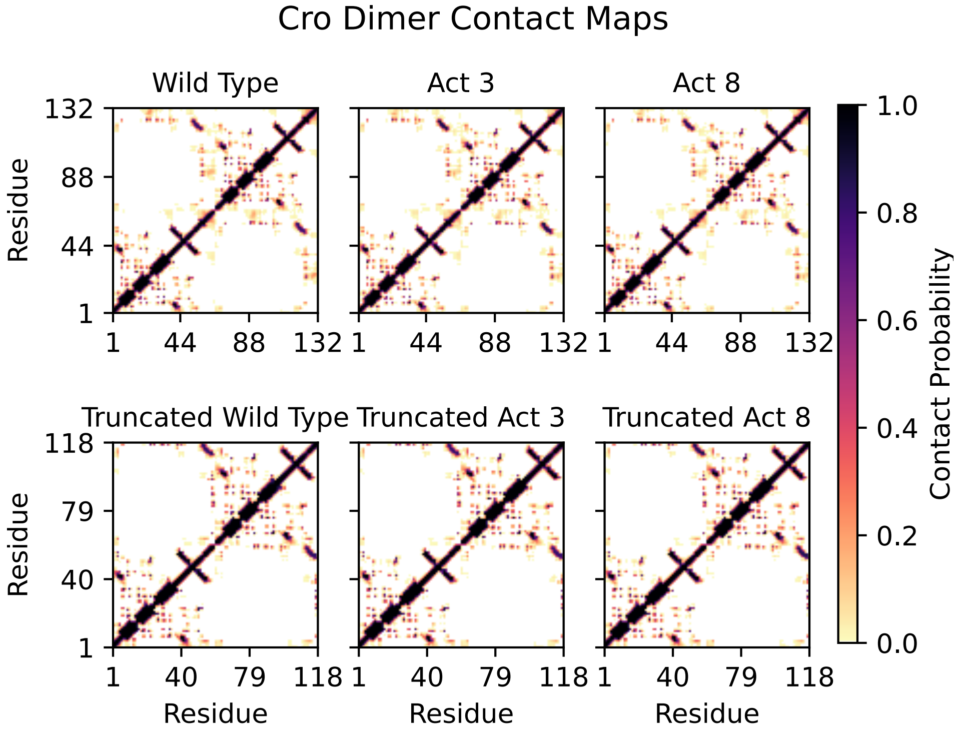

Figure S5: Contact maps of Cro dimer structures. Top: Residues 1-66 correspond to the first Cro subunit, and the remainder the second Cro subunit. Bottom: Residues 1-59 correspond to the first Cro subunit, and the remainder the second Cro subunit.

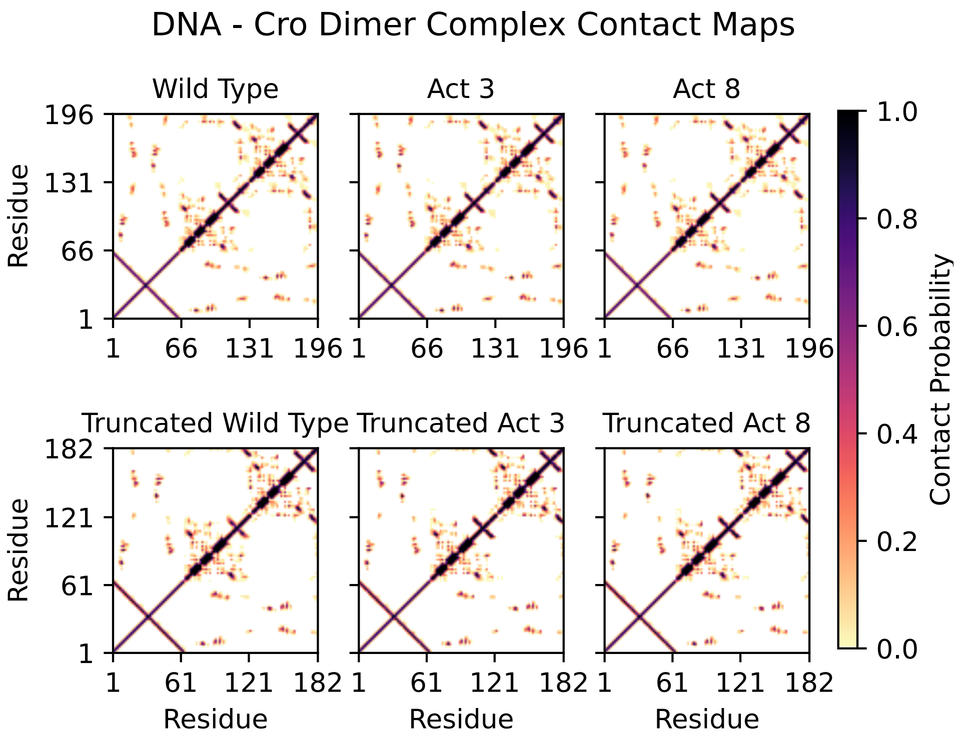

Figure S6: Contact maps of DNA-bound Cro dimer structures. Residues 1-64 represent DNA. Top: Residues 65-130 correspond to the first Cro subunit, and residues 131-196 correspond to the second Cro subunit. Bottom: Residues 65-123 correspond to the first Cro subunit, and residues 124-182 correspond to the second Cro subunit.

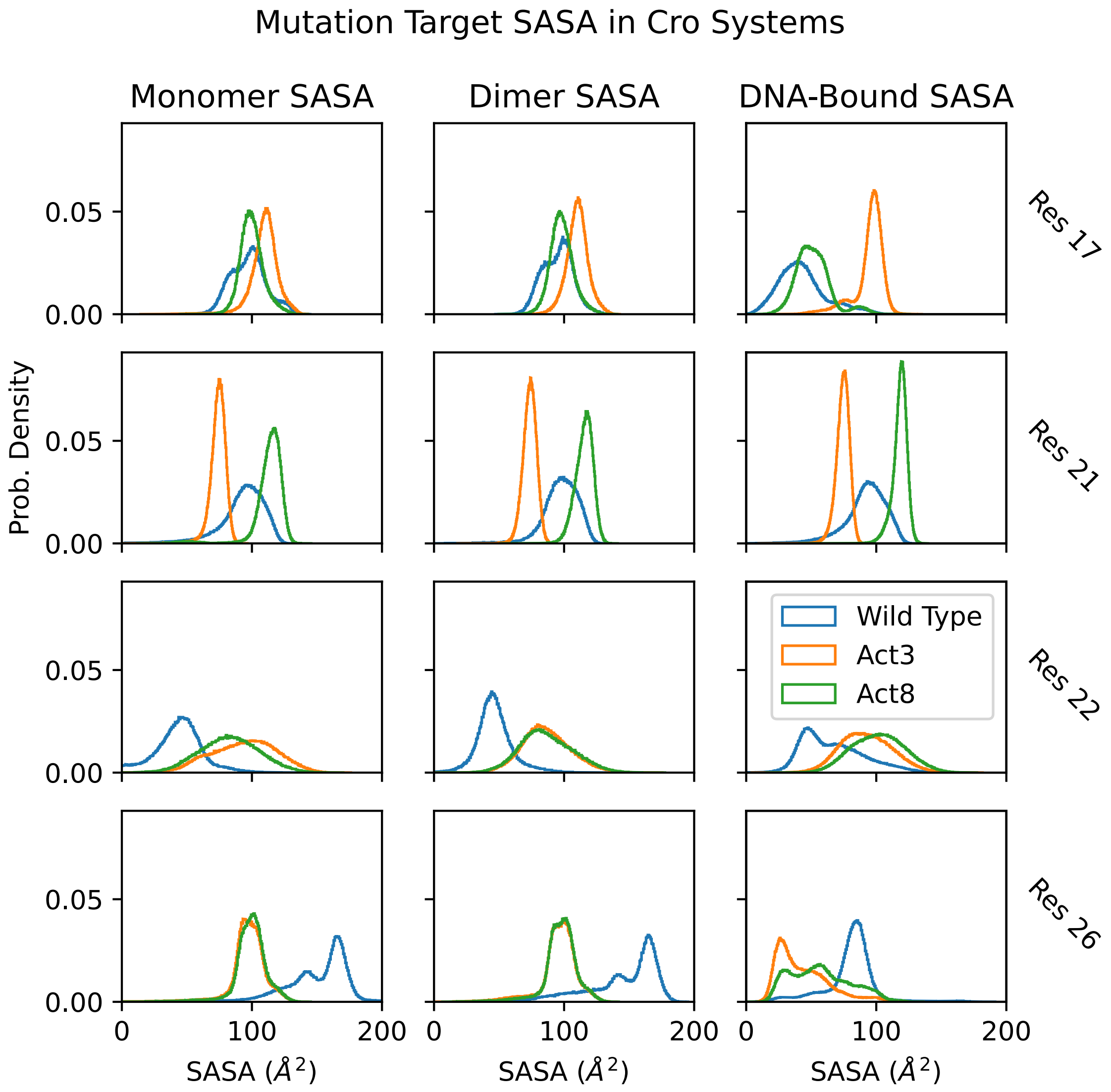

Figure S7: Solvent accessible surface area of mutation targets in Cro simulations. Differing amino acid geometries lead to changes in burial/accessibility. In systems with Cro dimers, distributions are taken from aggregating both subunits.

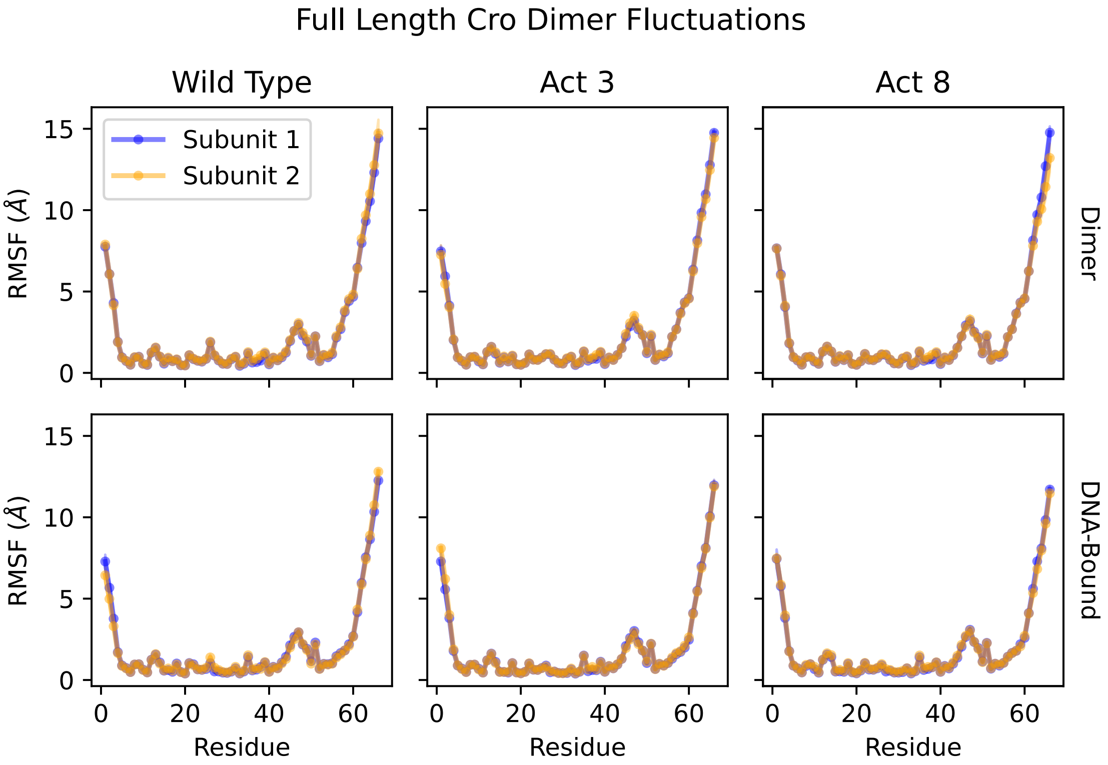

Figure S8: Root mean squared fluctuations of full-length, dimerized Cro subunits. Dimerized Cro subunits in similarly folded states experience symmetric fluctuations. Top row: Dimerized Cro. Bottom: DNA-bound Cro dimers.

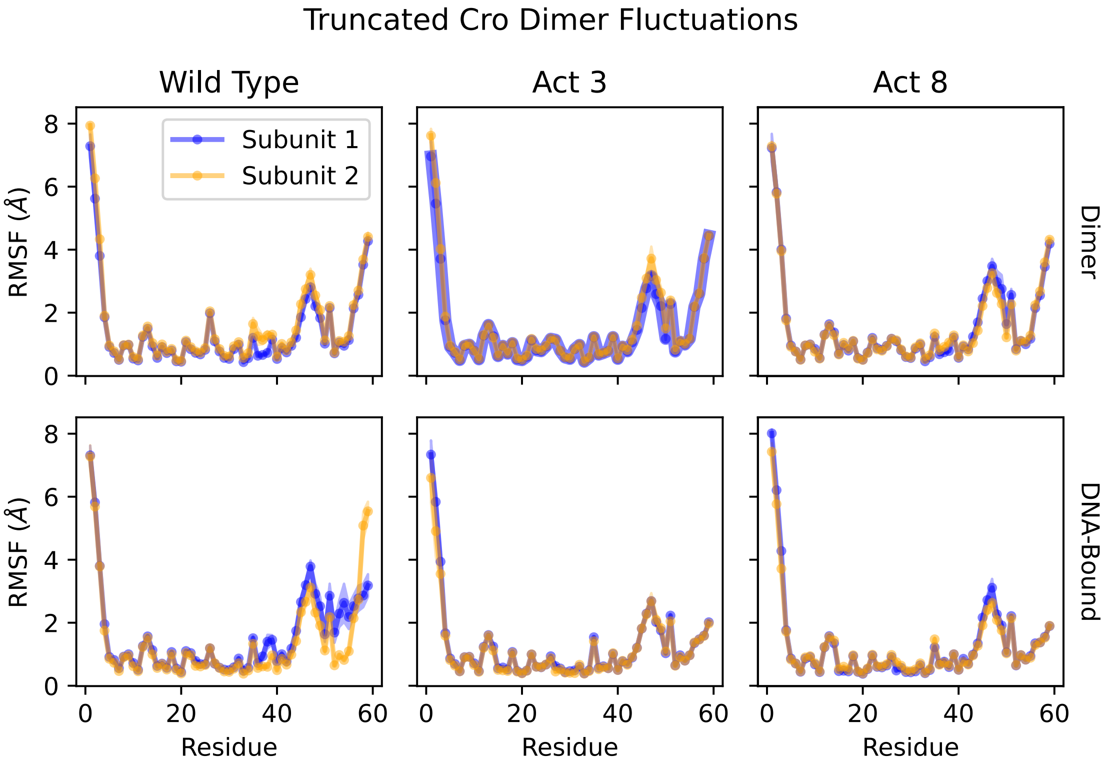

Figure S9: Root mean squared fluctuations of truncated, dimerized Cro subunits. Dimerized Cro subunits in similarly folded states experience symmetric fluctuations. Top row: Dimerized Cro. Bottom: DNA-bound Cro dimers.

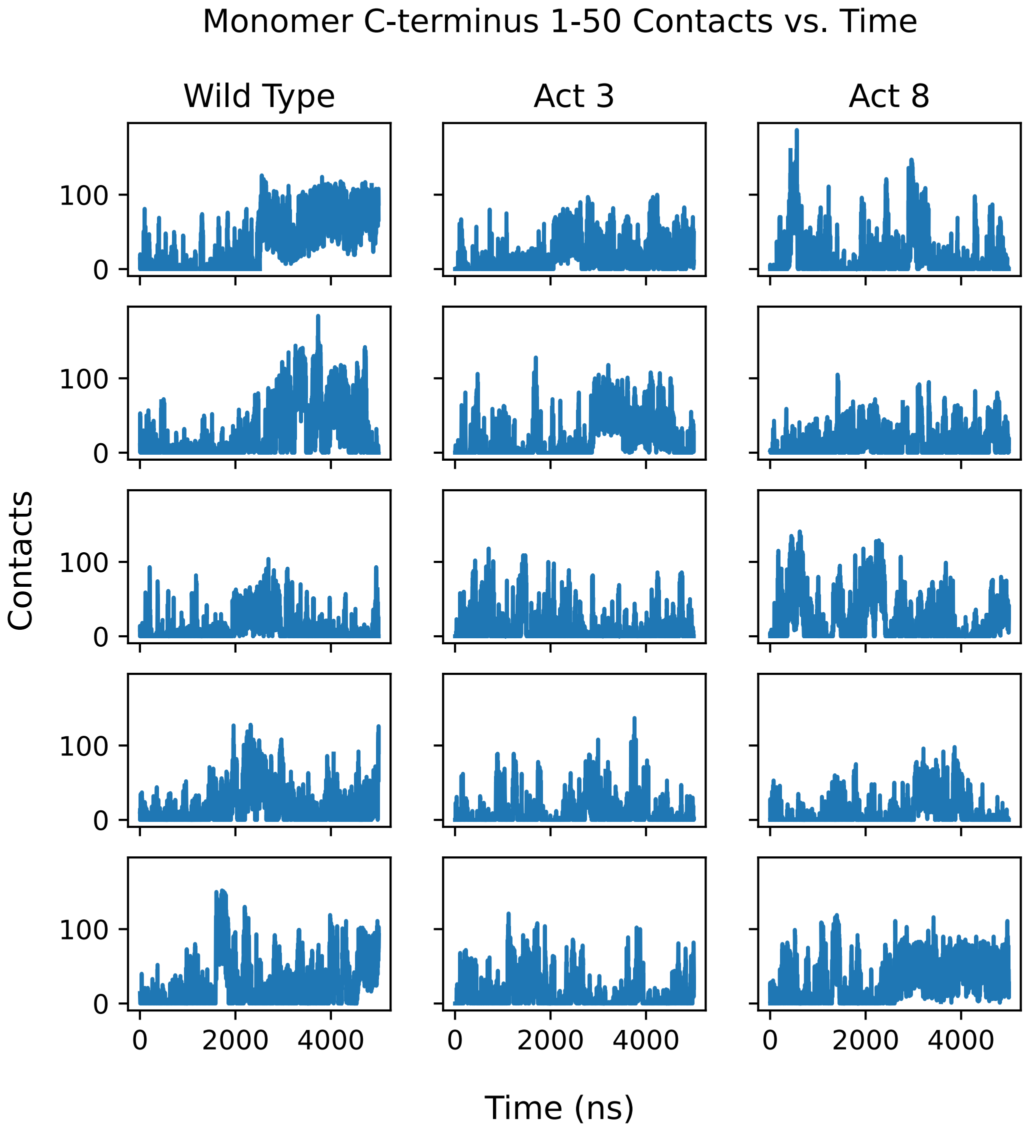

Figure S10: Contacts over time between residues 1-50 and residues 60-66 in full length Cro monomers. High variation within each run indicates transient contacts.

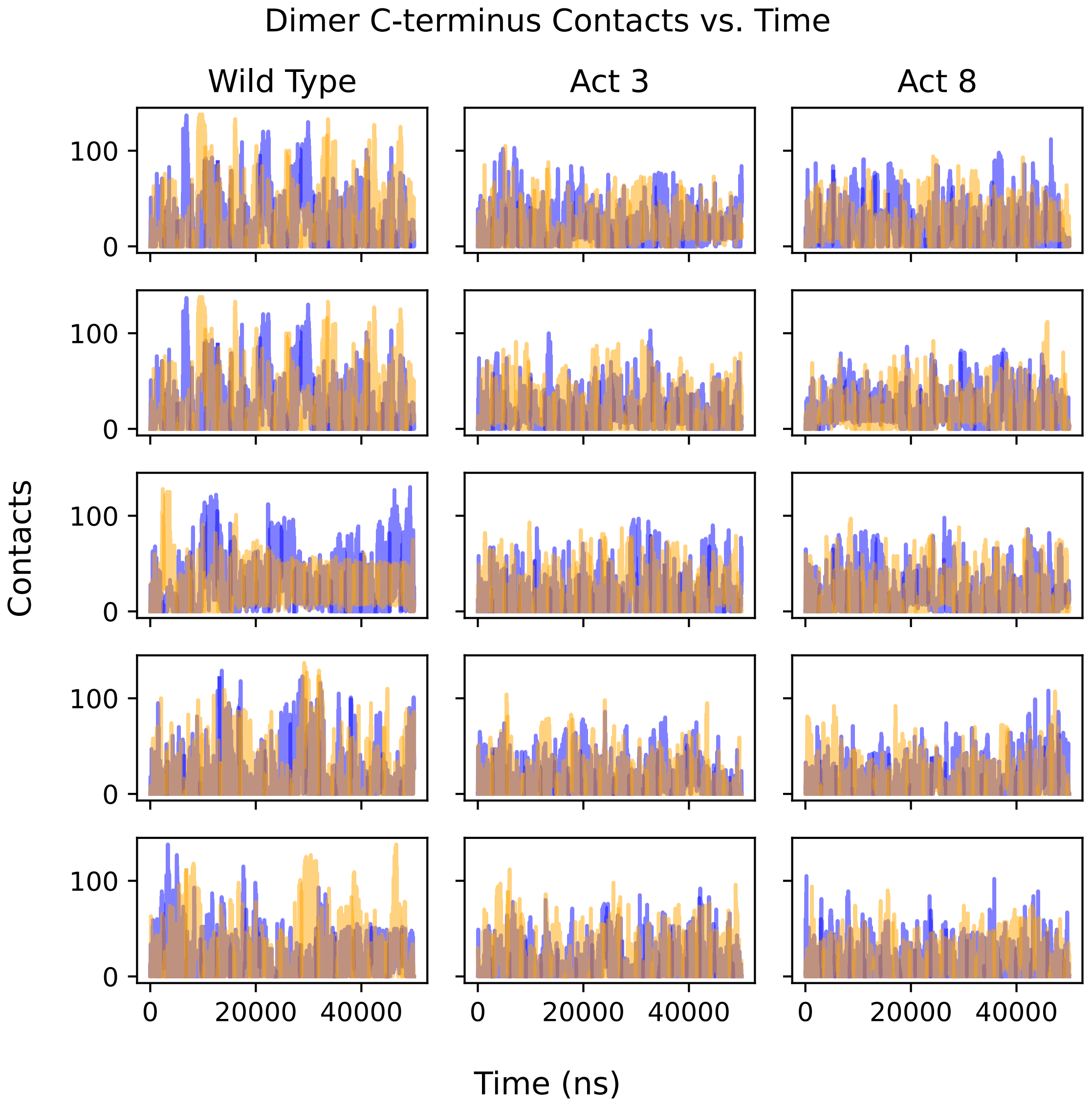

Figure S11: Contacts over time between residues 60-66 in each Cro subunit and residues 1-50 of both subunits in full length Cro dimers. High variation within each run indicates transient contacts.

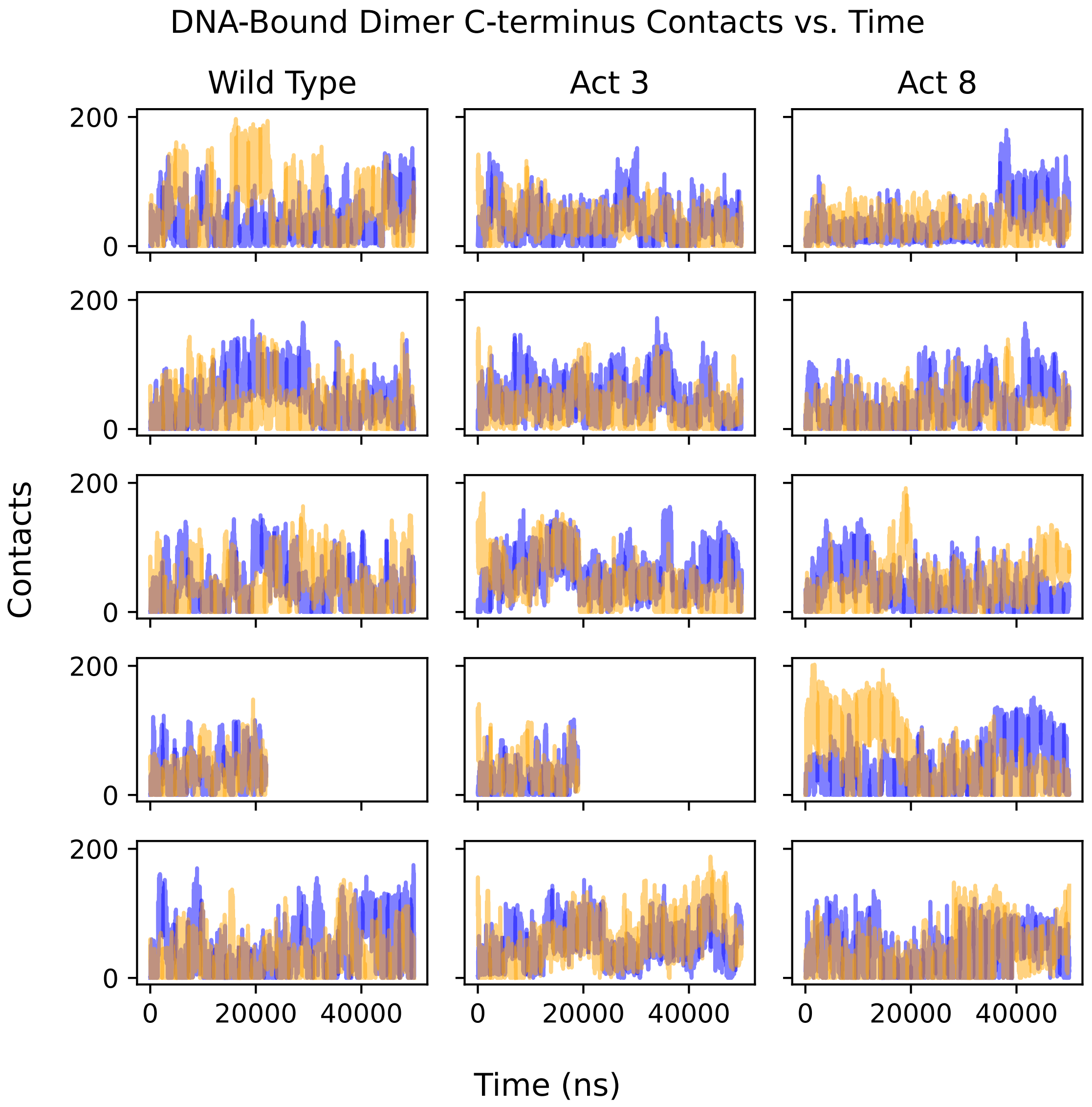

Figure S12: Contacts over time between residues 60-66 in each Cro subunit, DNA, and residues 1-50 of both subunits in full length Cro dimers. High variation within each run indicates transient contacts.

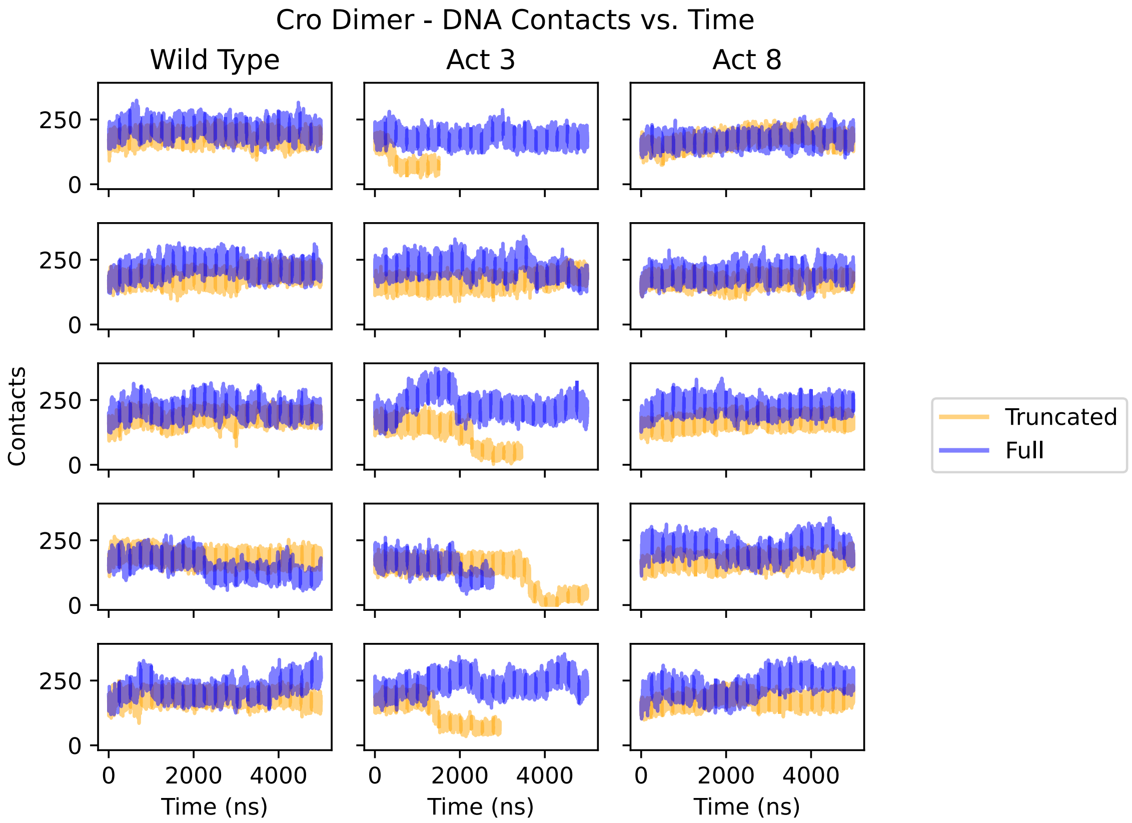

Figure S13: Contacts over time between both Cro subunits and DNA. Disassociation between one Cro subunit and DNA is associated with the drops seen in full length wild type's and Act3's fourth simulations, as well as the drops seen in all but the second simulation of truncated Act3. We did not continue most simulations after this disassociation.

Docking of wild-type Cro with RNA Polymerase Subunit α

Structure	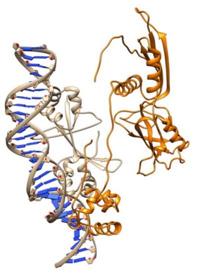	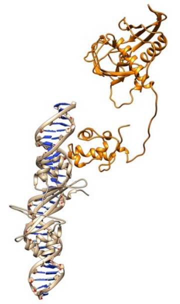	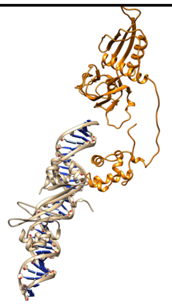	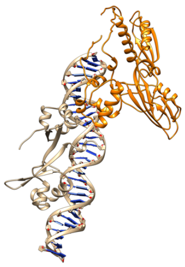	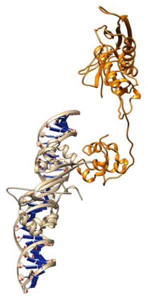
HADDOCK Score	-95.5 +/- 12.5	-87.5 +/- 1.5	-85.3 +/- 5.7	-83.6 +/- 4.9	-80.3 +/- 3.0
Z-Score	-1.7	-1.0	-0.8	-0.7	-0.4
Cluster Size (159 Total Structures)	6	20	11	15	24

Figure S14: Top 5 Results of HADDOCK molecular docking of wild-type Cro on DNA with RNA polymerase subunit α. Interactions between wild-type Cro and α are not known to biologically occur for recruiting RNAP.

Docking of wild-type Cro with RNA Polymerase Subunit σ^{70}

Structure	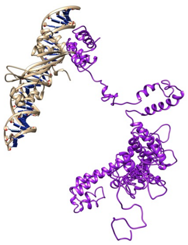	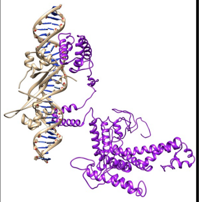	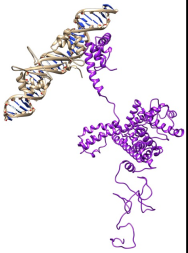	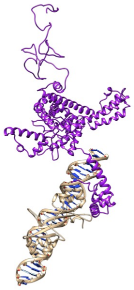	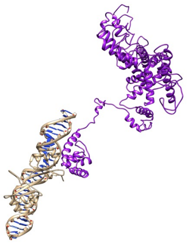
HADDOCK Score	-64.2 +/- 12.2	-48.1 +/- 16.1	-40.3 +/- 6.4	-20.1 +/- 10.3	-17.1 +/- 11.6
Z-Score	-2.2	-1.3	-0.8	0.3	0.4
Cluster Size (147 Total Structures)	39	8	11	7	12

Figure S15: Top 5 Results of HADDOCK molecular docking of wild-type Cro on DNA with RNA polymerase subunit σ. Interactions between wild-type Cro and σ are not known to biologically occur for recruiting RNAP. Nonetheless, HADDOCK metrics suggest a less favorable interaction than with subunit α.

Docking of Act3 Cro with RNA Polymerase Subunit α

Structure	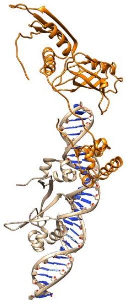	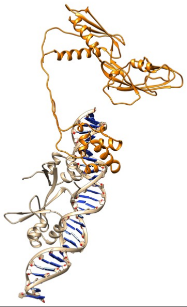	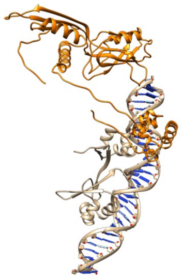	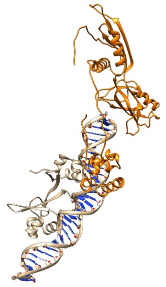	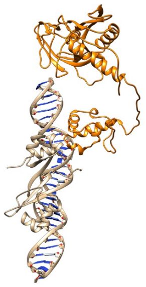
HADDOCK Score	-103.4 +/- 5.9	-86.4 +/- 5.2	-73.5 +/- 3.9	-73.3 +/- 8.4	-70.0 +/- 15.4
Z-Score	-1.9	-1.0	-0.3	-0.3	-0.1
Cluster Size (173 Total Structures)	94	16	9	5	10

Figure S16: Top 5 Results of HADDOCK molecular docking of Act3 Cro on DNA with RNA polymerase subunit α. Lower HADDOCK score, Z score, and high cluster size point to a more favorable interaction than the wild-type Cro.

Docking of Act3 Cro with RNA Polymerase Subunit σ^{70}

Structure	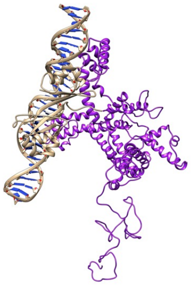	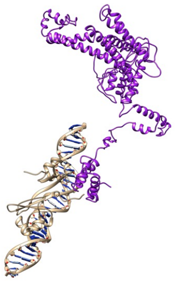	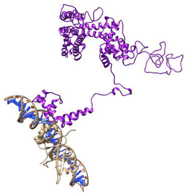	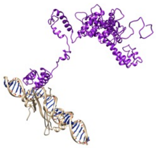	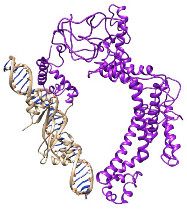
HADDOCK Score	-68.0 +/- 10.6	-45.6 +/- 10.8	-42.9 +/- 7.2	-18.0 +/- 11.9	-16.9 +/- 4.7
Z-Score	-1.6	-0.9	-0.8	0.0	0.0
Cluster Size (130 Total Structures)	17	70	14	10	5

Figure S17: Top 5 Results of HADDOCK molecular docking of Act3 Cro on DNA with RNA polymerase subunit σ. HADDOCK metrics suggest a less favorable interaction than with subunit α, comparable to wild-type Cro.

Docking of Act8 Cro with RNA Polymerase Subunit α

Structure	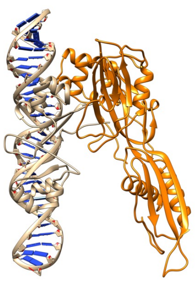	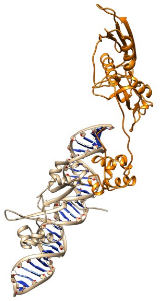	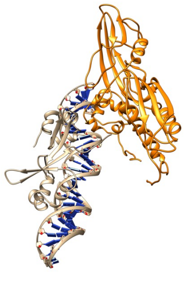	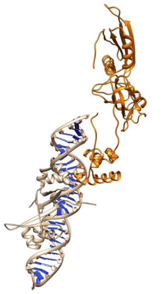	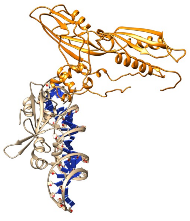
HADDOCK Score	-115.1 +/- 7.3	-105.0 +/- 5.5	-92.4 +/- 2.3	-79.8 +/- 4.4	-78.7 +/- 3.3
Z-Score	-1.6	-1.2	-0.7	-0.2	-0.1
Cluster Size (164 Total Structures)	17	58	26	20	10

Figure S18: Top 5 Results of HADDOCK molecular docking of Act8 Cro on DNA with RNA polymerase subunit α. Like Act3 Cro, the lower HADDOCK score, Z score, and higher cluster size point to a more favorable interaction than the wild-type Cro.

Docking of Act8 Cro with RNA Polymerase Subunit σ^{70}

Structure	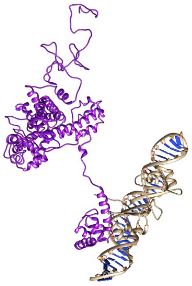	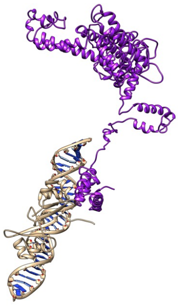	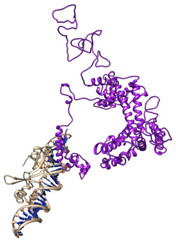	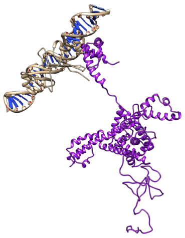	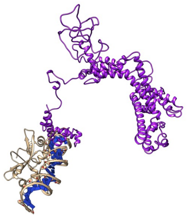
HADDOCK Score	-70.0 +/- 3.8	-64.0 +/- 9.2	-29.6 +/- 10.1	-29.0 +/- 11.6	-28.6 +/- 3.7
Z-Score	-2.0	-1.7	0.1	0.2	0.2
Cluster Size (134 Total Structures)	41	17	4	10	7

Figure S19: Top 5 Results of HADDOCK molecular docking of Act8 Cro on DNA with RNA polymerase subunit σ. HADDOCK metrics suggest a less favorable interaction than with subunit α, comparable to wild-type Cro.
